# Adjuvant chemotherapy for muscle-invasive bladder cancer: a systematic review and network meta-analysis of randomized clinical trials

**DOI:** 10.18632/oncotarget.20979

**Published:** 2017-09-18

**Authors:** Hyung Suk Kim, Chang Wook Jeong, Cheol Kwak, Hyeon Hoe Kim, Ja Hyeon Ku

**Affiliations:** ^1^ Department of Urology, Dongguk University Ilsan Medical Center, Goyang, Korea; ^2^ Department of Urology, Seoul National University Hospital, Seoul, Korea

**Keywords:** bladder cancer, muscle-invasive, chemotherapy, cystectomy, systematic review

## Abstract

Although adjuvant chemotherapy (ACH) is widely used in clinical practice for the management of muscle-invasive bladder cancer (MIBC), a consensus has yet to be established on which ACH regimen is the most effective for improving postoperative survival. In this study, we aimed to systematically assess the optimal ACH regimen for improving survival outcomes in patients treated with radical cystectomy (RC) for MIBC. A comprehensive literature search was conducted in the PubMed, Embase, and the Cochrane Library databases for all articles published until December 2016 in accordance with the Preferred Reporting Items for Systematic Reviews and Meta-analyses (PRISMA) guidelines. The study end-points were progression-free survival (PFS) and overall survival (OS). A direct pairwise meta-analysis was conducted by pooling the studies that compared RC with ACH and RC alone, and the results are presented as a pooled hazard ratio (HR) with a 95% confidence interval (CI). A Bayesian network meta-analysis was adopted for indirect comparisons among various ACH regimens, and the outcomes are presented as HRs with 95% credible intervals (CrI). The eleven randomized controlled trials ultimately selected for the current analysis comprised of 1,546 patients with 49 to 327 subjects per study. Based on the pairwise meta-analysis, the use of ACH showed significantly better PFS (HR, 0.64; 95% CI, 0.49–0.85) and OS (HR, 0.79; 95% CI, 0.68–0.92) than RC alone. In the network meta-analysis, the gemcitabine/cisplatin/paclitaxel (GCP) combination was the only ACH regimen associated with significant improvement in both the PFS (HR, 0.38; 95% CrI, 0.25–0.58) and OS (HR, 0.38; 95% CrI 0.22–0.65). ACH following RC for MIBC may therefore contribute to improved PFS and OS. In particular, the GCP combination may be the optimal ACH regimen for improving postoperative survival outcomes. Additional well-designed, large scale, prospective, randomized trials are still required to establish the optimal ACH regimen in MIBC patients.

## INTRODUCTION

Muscle-invasive bladder cancer (MIBC), which accounts for 20% to 30% of all bladder cancers at the initial diagnosis, is primarily treated with radical cystectomy (RC) combined with bilateral pelvic lymph node dissection (PLND) [1]. A substantial number of patients with localized MIBC may be completely cured by RC alone, with a 5-year recurrence-free survival (RFS) rate of more than 80% [1]. However, in spite of this potentially curative surgical treatment, some MIBC patients experience locoregional or distant disease recurrence postoperatively. In cases of locally advanced MIBC, including pT3–4 tumor or lymph node positive (N+) disease, the 5-year RFS and overall survival (OS) rates after RC are 35 to 60% and 25% to 50%, respectively [1, 2].

These low survival outcomes in locally advanced MIBC may be due to systemic occult micrometastases at the time of RC, which cannot be detected by preoperative imaging studies [3]. Also, distant recurrence of bladder cancer is more frequent than locoregional recurrence [1, 4]. These findings suggest that RC alone may be insufficient to completely control the disease and that theadditional use of systemic therapy should be considered in the majority of patients with locally advanced MIBC.

To improve the survival outcome of MIBC patients by eradicating micrometastatic disease, the use of perioperative (neoadjuvant or adjuvant) systemic chemotherapy in conjunction with RC has been intensively investigated. The survival advantages of neoadjuvant chemotherapy (NACH) have been proven by several randomized controlled trials (RCTs) and meta-analyses, which have reported a 5% improvement in OS and a 9% improvement in disease-free survival [5–8]. Therefore, depending upon the current international guidelines [3], the use of cisplatin-based NACH is recommended as level of evidence I in patients with non-metastatic MIBC (cT2-T4a). In light of the observed survival benefits of NACH, several clinical trials and meta-analyses investigating the use of adjuvant chemotherapy (ACH) after RC in advanced bladder cancer have been conducted [9–16]. However, the evidence supporting the utility of ACH for the management of MIBC remains inadequate due to study limitations; these limitations include the difficulty of designing prospective studies with a small sample size and patient dropouts due to poor general condition and diminished renal function postoperatively [2, 16]. Consequently, there is no evidence-based consensus regarding which ACH regimen should be used clinically.

In the present study, we aimed to evaluate the efficacy of ACH and determine the optimal ACH regimen associated with significant improvement in survival outcomes in MIBC patients who underwent RC. To accomplish this, we performed a systematic review of the published literature and a network meta-analysis of the available data.

## RESULTS

### Literature search results

**Figure 1:**
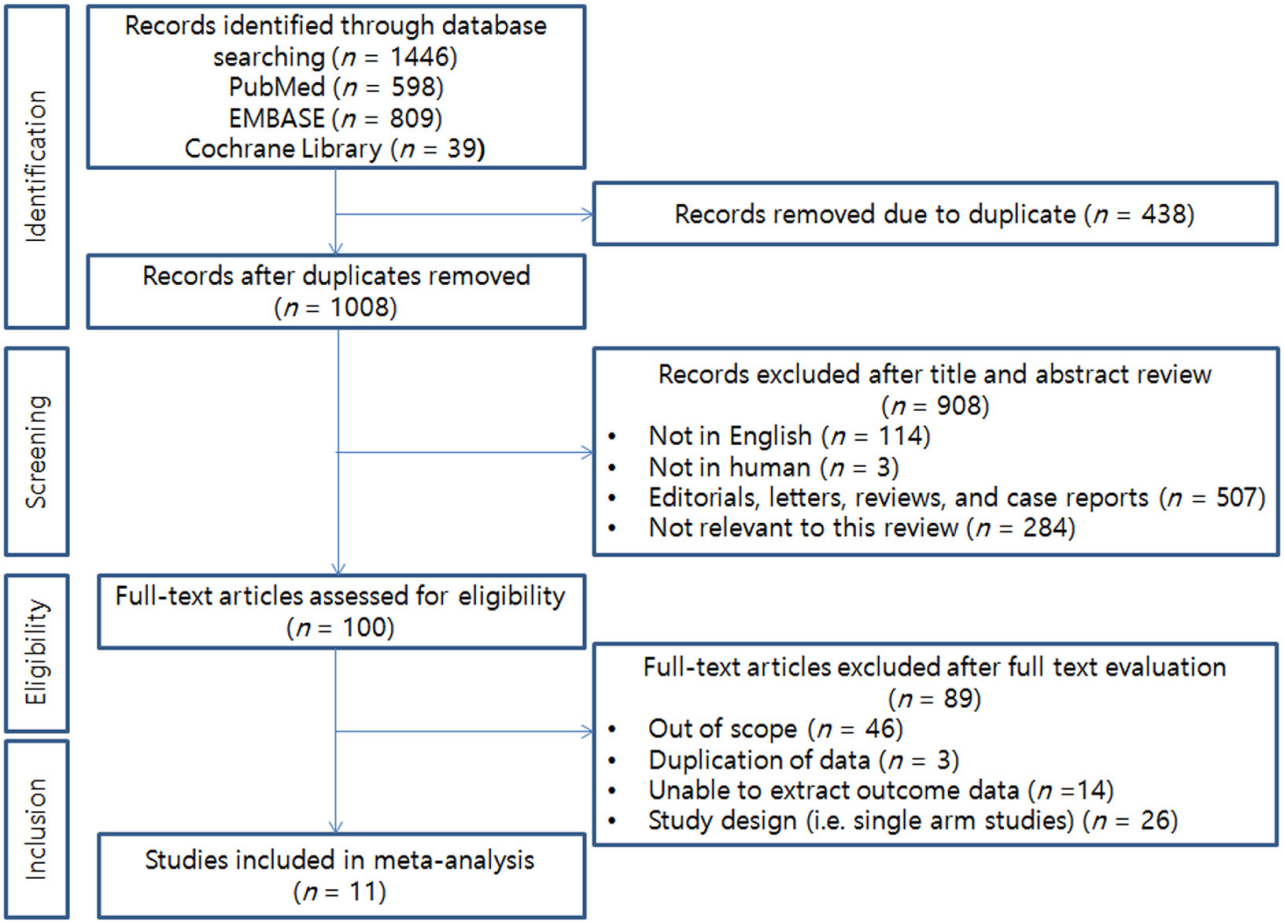
PRISMA flow diagram illustrating the search strategy used for the network meta-analysis

The initial database searches yielded 1,446 articles, of which 438 were excluded for being duplicate publications. Following the review of titles and abstracts, we excluded another 908 articles. A total of 100 articles remained for full-text review. In accordance with all previously mentioned inclusion criteria, a total of 11 RCTs conducted between 1991 and 2015 were ultimately included in the current meta-analysis [9, 11–15, 17–21]. The PRISMA flow chart describing the literature search and selection of studies is shown in Figure 1.

### Overview of included studies

### Study characteristics

Table 1 presents detailed information of each included study, all of which were prospective RCTs. Seven studies were performed in Europe [11, 12, 14, 18–21], three studies were conducted in the United States [9, 13, 17], and the remaining study was multinational from Europe and Canada [15]. Two trials were included as conference abstracts without full text publications [20, 21]. The recruitment period of patients ranged from 1980 to 2008. The distribution of patients to control and case (ACH) groups utilized a nearly 1:1 randomization in each study, ranging from 23 to 143 subjects per group. Because we only included prospective RCTs, most of the studies investigated in this review satisfied all of the evaluation criteria. The quality scale ranged from 4 to 6, and 7 of the 11 studies showed a quality scale of 6, implying that most of the included studies were well-designed and of high quality. Additional characteristics of the included studies are listed in Table 1.

**Table 1:** Study characteristics of the eligible studies

Study	Year	Country	Recruitment period	Total patients (ITT)	Median age, range (years)	No. of gender (male/female)	Quality scale
Skinner [17]	1991	USA	1980–1988	Control: 52 ACH: 50	Control: 62, 30–73 ACH: 61, 22–75	Control: 35/12 ACH: 34/10	6
Studer [18]	1994	Switzerland	1984–1989	Control: 45 ACH: 46	Control: 61, 41–65 ACH: 64, 54–73	Control: 27/13 ACH: 30/7	6
Freiha [9]	1996	USA	1986–1993	Control: 28 ACH: 27	Control: 64 (mean), 49–78 ACH: 59 (mean), 40–76	Control: 23/2 ACH: 22/3	6
Bono [19]	1997	Italy	1984–1987	Control: 47 ACH: 43	NA	NA	4
Otto [20]	2001	Germany	1993–1999	Control: 53 ACH: 55	NA	NA	4
Lehmann [11]	2005	Germany	1994–2000	CM: 163 MVEC: 164	CM: 60.2 MVEC: 60.7	CM: 123/40 MVEC: 134/30	6
Lehmann [12]	2006	Germany	1987–1990	Control: 23 ACH: 26	Control: 62.7 ACH: 58.8	Control: 19/4 ACH: 22/4	6
Paz-Ares [21]	2010	Spain	2000–2007	Control: 74 ACH: 68	63 overall	NA	5
Stadler [13]	2011	USA	1997–2006	Control: 56 ACH: 58	NA	Control: 47/9 ACH: 51/7	5
Cognetti [14]	2012	Italy	2001–2007	Control: 92 ACH: 102	Control: 63, 36–75 ACH: 64: 38–75	Control: 75/11 ACH: 90/7	6
Sternberg [15]	2015	Europe and Canada	2002–2008	Control: 143 ACH: 141	Control: 61, 37–76 ACH: 61, 35–82	Control: 112/27 ACH: 114/27	6

ITT: intention-to-treat, ACH: adjuvant chemotherapy, CM: cisplatin and methotrexate, NA: not available, MVEC: methotrexate, vinblastine, epirubicin and cisplatin.

### Treatment characteristics

Details concerning the treatment characteristics of the eligible studies are summarized in Table 2. In general, RC involved complete extirpation of the entire bladder, prostate, and seminal vesicles in men and removal of the anterior pelvic organs in women, including the bladder, uterus, and a portion of the anterior vagina. PLND implied bilaterally full dissection of the lymph nodes bordered by the internal iliac area, external iliac area, common iliac bifurcation, and abdominal aortic bifurcation area. Any evidence of macroscopic (grossly and palpable unresected lymph nodes) or microscopic (tumor positive margins of the specimen) disease was considered to be an exclusion criterion in most of the studies [9, 11–15, 17–19]. The number of removed lymph nodes varied among the included studies, and detailed information regarding urinary diversion was not identified in every study. The pathologic stages in most trials included muscle-invasive or locally advanced (pN+) disease without distant metastases. Assessed chemotherapy regimens consisted of the following: a cisplatin-based combination, including cisplatin, Adriamycin (doxorubicin), and cyclophosphamide (CAP) [17]; cisplatin and methotrexate (CM) [19]; cisplatin, methotrexate, and vinblastine (CMV) [9]; methotrexate, vinblastine, Adriamycin, and cisplatin (MVAC) [13, 15]; methotrexate, vinblastine, epirubicin, and cisplatin (MVEC) [11, 12, 20]; gemcitabine, cisplatin, and paclitaxel (GCP) [21]; and gemcitabine and cisplatin (GC) [14, 15]. Only one trial investigated adjuvant cisplatin monotherapy [18]. The dosages of each chemotherapeutic agent were similar when specific ACH regimens (MVAC, MVEC, and GC) were used. The number of cycles of ACH ranged from 3 to 4 in most studies.

**Table 2:** Treatment characteristics of the eligible studies

Study	Pathologic stage	Chemotherapy regimens	Chemotherapy dosage (mg/m^2^)	No. of planned cycles	Median follow-up, range (months)
Skinner [17]	T3-4, Nany, M0	CAP	C 100, A 60, P 600	4	168 overall
Studer [18]	T1-4a, M0	C	C 90	3	69 overall
Freiha [9]	T3b-4, Nany, M0	CMV	C 100, M 30, V 4	4	62, 26-94 overall
Bono [19]	T2-4a, N0, M0	CM	C 70, M 40	4	69 overall
Otto [20]	T3, N1-2, M0	MVEC	M 30, V 3, E 45, C 70	3	3.62 yr overall
Lehmann [11]	T3-4a, Nany, M0	CM vs MVEC	C 70, M 40 M 30, V 3, E 45, C 70	3	42 overall
Lehmann [12]	T3-4a, Nany, M0	MVAC or MVEC	M 30, V 3, A 40, C 70 M 30, V 3, E 45, C 70	3	Control: 57 ACH: 54
Paz-Ares [21]	T3-4, Nany, M0	GCP	G 1000, C 70, P 80	4	30, 1-95 overall
Stadler [13]	T1-2, N0, M0	MVAC	NA	3	5.4 yr overall
Cognetti [14]	T2-4, Nany, M0	GC	G 1000, C 70	4	35, 15-57 (IQR) overall
Sternberg [15]	T3-4, Nany, M0	(high dose) MVAC or GC	M 30, V 3, A 30, C 70 G 1000, C 70	4	Control: 7.2 yr, 5.6-8.7 yr (IQR) ACH: 7 yr, 5.2-8.7 yr (IQR)

CAP: cisplatin, doxorubicin and cyclophosphamide, C: cisplatin, CMV: cisplatin, methotrexate and vinblastine, CM: cisplatin and methotrexate, MVEC: methotrexate, vinblastine, epirubicin and cisplatin, MVAC: methotrexate, vinblastine, doxorubicin and cisplatin, ACH: adjuvant chemotherapy, GCP: gemcitabine, cisplatin and paclitaxel, GC: gemcitabine and cisplatin, IQR: interquartile range.

### Pairwise meta-analysis

**Figure 2:**
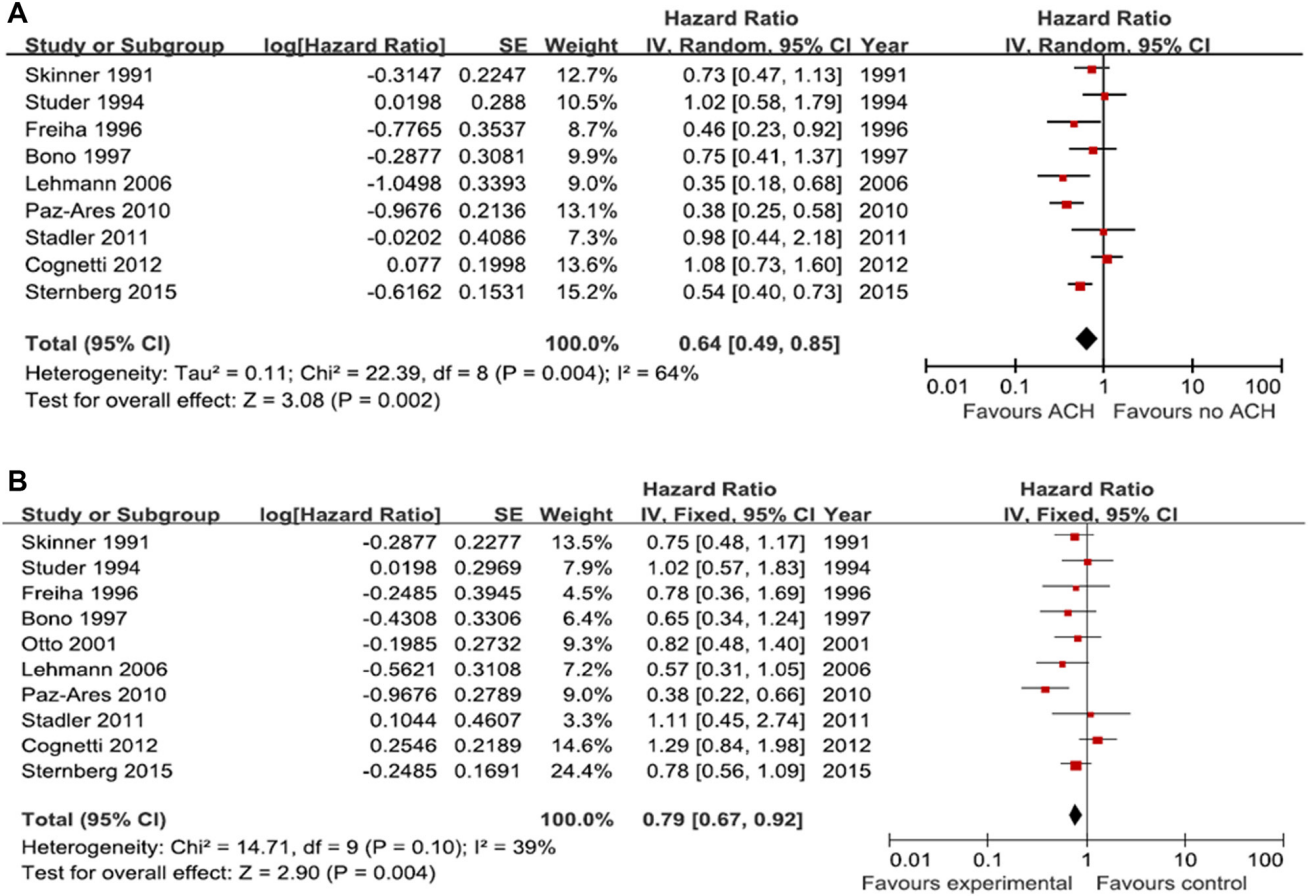
Forest plots of prognosis with adjuvant chemotherapy The horizontal lines correspond to the study-specific hazard ratio and 95% confidence interval, respectively. The area of the squares reflects the study-specific weight. The diamond represents the results for pooled hazard ratio and 95% confidence interval. (**A**) progression-free survival; (**B**) overall survival.

A total of 9 studies including 1,111 patients, were available for the meta-analysis of progression-free survival (PFS). The pooled analysis of PFS indicated that ACH was significantly associated with better PFS outcomes than controls (hazard ratio [HR], 0.64; 95% confidence interval [CI], 0.49–0.85; Figure 2A). Significant heterogeneity among the included studies for PFS was observed (*p*=0.004; I^2^ = 64%). The pooled analysis of OS was based on ten publications involving 1,219 patients. The pooled HR (95% CI) was 0.79 (0.67–0.92), which suggested favorable OS outcomes for patients who received ACH compared to controls (Figure 2B). There was no significant heterogeneity among included studies for OS (*p* = 0.10; I^2^ = 39%).

Sensitivity analysis was conducted to evaluate the influence of individual studies on the overall meta-analysis results by omitting one study at a time. Omission of any study made no significant difference, demonstrating that our results were statistically reliable (data not shown).

We could find no strong evidence suggesting publication bias by graphical inspection in the pairwise meta-analyses of both PFS and OS. Funnel plots for publication bias for PFS and OS demonstrated a certain degree of symmetry (Supplementary Figure 1). Moreover, the Begg's and Egger's tests revealed that there was no statistical evidence of publication bias in the pairwise meta-analysis of PFS and OS (all *p*-values > 0.05; Supplementary Figure 1).

### Bayesian framework network meta-analysis

The networks of the indirect comparisons among multiple ACH regimens, in terms of PFS and OS, are depicted in Supplementary Figures 2 and 3. A fixed effects model was selected because the Deviance Information Criteria (DIC) for the fixed effects model was lower than that for the random effects model.

### Primary endpoint: PFS

**Figure 3:**
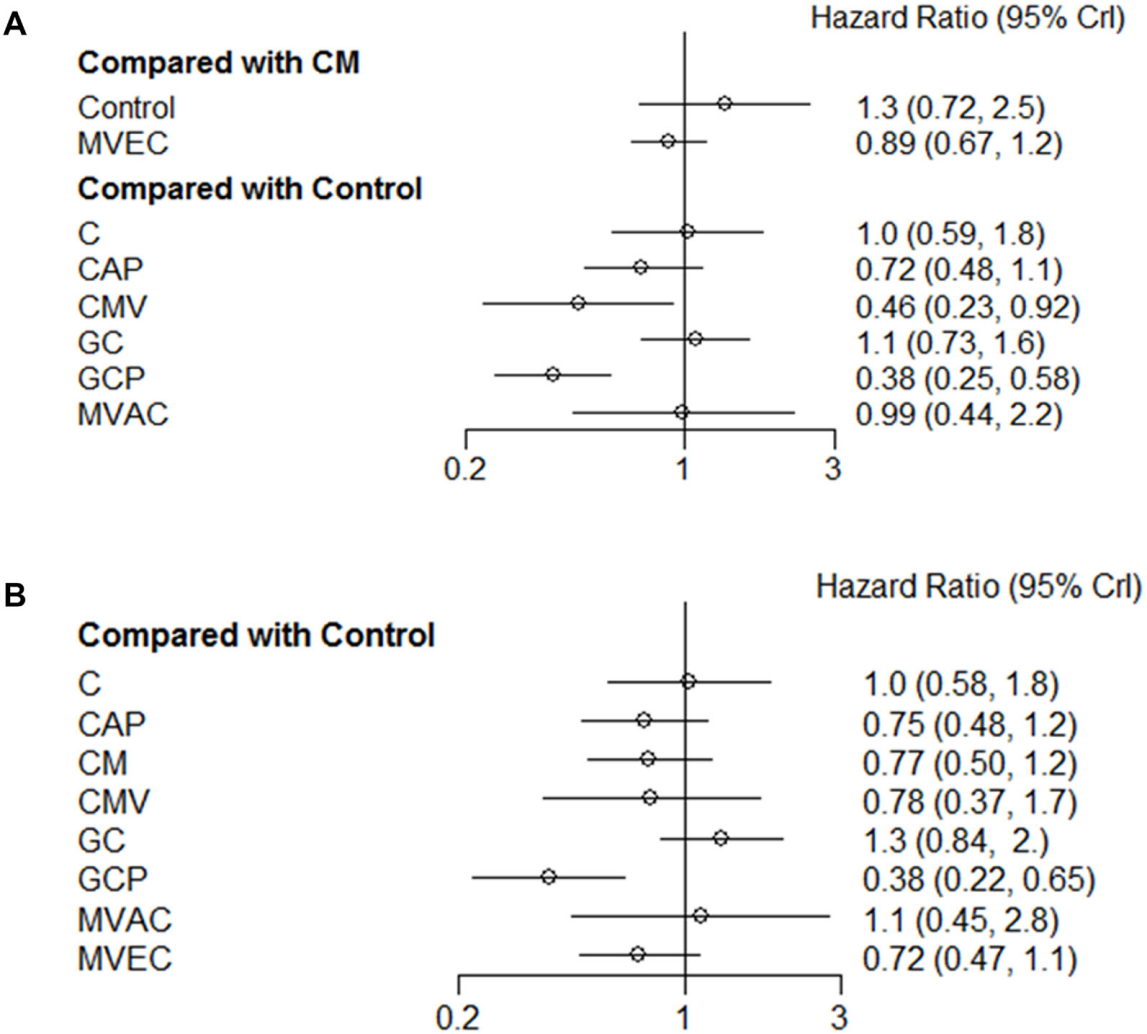
Pooled hazard ratio and 95% credible intervals for the network meta-analysis of survival outcomes (**A**) progression-free survival; (**B**) overall survival. C: cisplatin; CAP: cisplatin, doxorubicin, and cyclophosphamide; CM: cisplatin and methotrexate; CMV: cisplatin, methotrexate, and vinblastine; GC: gemcitabine and cisplatin; GCP: gemcitabine, cisplatin, and paclitaxel; MVAC: methotrexate, vinblastine, doxorubicin, and cisplatin; MVEC: methotrexate, vinblastine, epirubicin, and cisplatin; CrI: credible interval.

**Figure 4:**
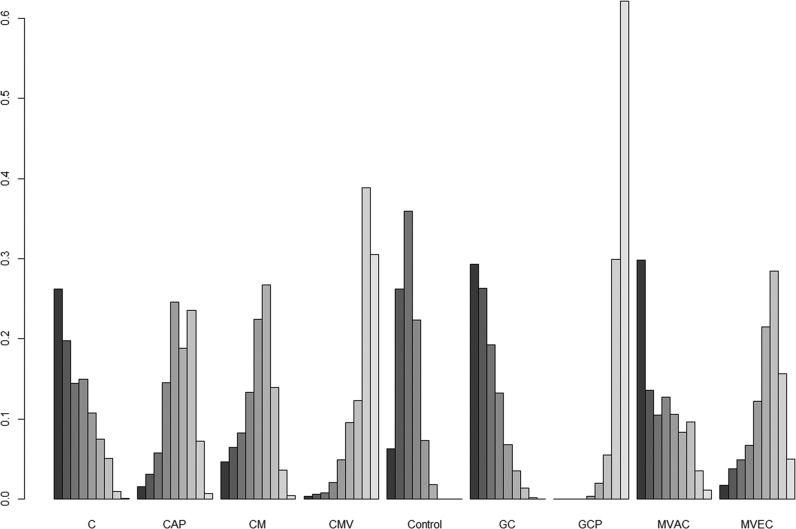
Rankograms for adjuvant chemotherapy network of progression-free survival The size of each bar corresponds to the probability of each treatment having a specific rank. C: cisplatin; CAP: cisplatin, doxorubicin, and cyclophosphamide; CM: cisplatin and methotrexate; CMV: cisplatin, methotrexate, and vinblastine; GC: gemcitabine and cisplatin; GCP: gemcitabine, cisplatin, and paclitaxel; MVAC: methotrexate, vinblastine, doxorubicin, and cisplatin; MVEC: methotrexate, vinblastine, epirubicin, and cisplatin.

The results for network meta-analysis of PFS are described in Figure 3A. Among the ACH regimens examined, the CMV (HR, 0.46; 95% credible interval [CrI] 0.23–0.92) and GCP (HR, 0.38; 95% CrI 0.25–0.58) regimens significantly correlated with favorable PFS compared with controls. There were no significant PFS differences between other regimens (cisplatin, CAP, GC, MVAC, and CM) and controls. Figure 4 shows the ranking results of 9 different ACH regimens (including controls) in terms of PFS benefit. The GCP and CMV regimens had a high probability of being ranked first or second, respectively; the GC regimen was most likely to be the worst ranked, and it was inferior to controls. The rankings of these ACH regimens are similarly presented in Supplementary Table 1 and Figure 4A.

### Secondary endpoint: OS

**Figure 5:**
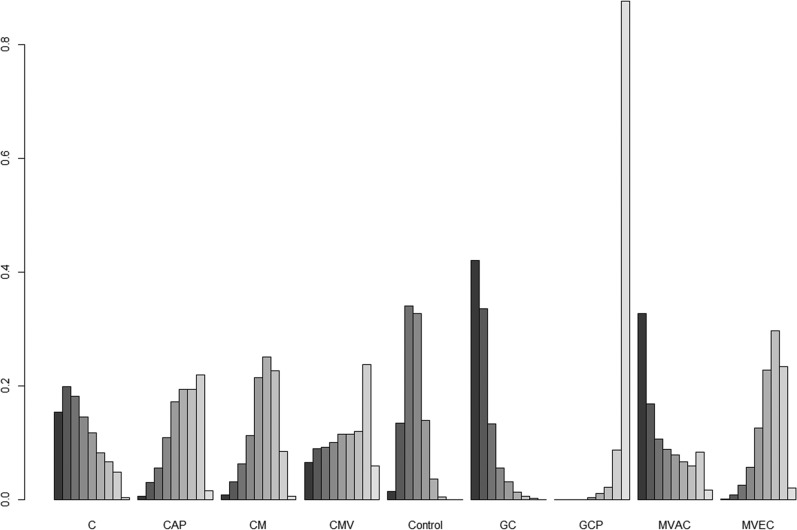
Rankograms for adjuvant chemotherapy network of overall survival The size of each bar corresponds to the probability of each treatment having a specific rank. C: cisplatin; CAP: cisplatin, doxorubicin, and cyclophosphamide; CM: cisplatin and methotrexate; CMV: cisplatin, methotrexate, and vinblastine; GC: gemcitabine and cisplatin; GCP: gemcitabine, cisplatin, and paclitaxel; MVAC: methotrexate, vinblastine, doxorubicin, and cisplatin; MVEC: methotrexate, vinblastine, epirubicin, and cisplatin.

Figure 3B shows the network meta-analysis results of OS. Compared to controls, only the GCP regimen (HR, 0.38; 95% CrI 0.22–0.65) was significantly associated with better OS. As for OS benefits, rankograms depicted in Figure 5 indicate that the GCP regimen had a higher probability of being ranked first than any other ACH regimen; the GC and MAVC regimens were likely to be the worst ranked, showing an inferior rank to controls. Similar results are presented in Supplementary Table 2 and Figure 4B.

## DISCUSSION

Patients with locally advanced MIBC (pT3-4 or N+ disease) are at high risk for recurrence or progression after RC. Because bladder cancer is generally chemosensitive, ACH has been widely used in locally advanced bladder cancer to control micrometastatic disease and to improve postoperative survival [22–24]. There are several potential advantages of treating MIBC with RC and ACH. By performing RC preferentially, it is possible to avoid any delay in curative local treatment in chemoresistant patients. Final pathological analysis of RC specimens allows clinicians to select patients at highest risk for recurrence who are most likely to benefit from ACH. There is no possibility of postoperative intravesical recurrence following RC, and micrometastases can be treated when the tumor burden is low by using systemic chemotherapy in the immediate post-RC period [25].

Several RCTs and meta-analyses have assessed the efficacy of various ACH regimens in MIBC in terms of survival benefit, however, the reported results have been inconsistent. Skinner et al. reported the first prospective randomized trial on ACH in MIBC using the CAP regimen [17]. They randomized 91 patients with pT3-4 or N+ disease to either the ACH (*n* = 47) or observation (*n* = 44) group; median survival duration was significantly extended in the ACH group relative to the observation group (4.3 vs. 2.4 years; *p* = 0.0062), and the 3-year disease free survival rate was also significantly better in the ACH group (70% vs. 46%; *p* = 0.001). Likewise, Freiha et al. evaluated the efficacy of the CMV regimen in 50 locally advanced MIBC patients [9]. Using a 1:1 randomization to assign patients to either the ACH or observation group, a significant PFS benefit was observed in the ACH group (37 months vs. 12 months; *p* = 0.01), but no significant OS benefit was found between the two groups (63 months vs. 36 months; *p* = 0.32). An early meta-analysis of 6 RCTs with 491 patients reported a 25% relative reduction (HR, 0.75; 95% CI, 0.60–0.96) in the risk of death for patients who received RC and ACH compared with RC alone [10]. Furthermore, a recent updated meta-analysis including 9 RCTs (n=945) revealed that RC with cisplatin-based ACH in MIBC was associated with a 23% decrease in all-cause mortality (HR, 0.77; 95% CI, 0.59–0.99) and a 34% decrease in cancer-related mortality (HR, 0.66; 95% CI, 0.45–0.91) compared with RC alone [16]. In contrast, Studer et al. investigated the efficacy of cisplatin monotherapy in advanced bladder cancer patients, randomizing 77 patients (40 in the ACH arm and 37 in the no-ACH arm), and demonstrated that there was no significant difference in RFS or OS between the two groups [18]. Similarly, several other RCTs using various combination ACH regimens (CM, MVEC, MAVC, and GC) have consistently reported no meaningful differences in postoperative survival outcomes compared with RC alone [13–15, 19, 20].

The trials evaluating ACH in MIBC had several limitations. First, it was often difficult or impossible for patients to receive planned systemic chemotherapy after RC owing to poor general condition, renal function deterioration, or complications postoperatively. For example, in a RCT conducted by Skinner et al., 25% of patients allocated to the ACH group did not receive adjuvant chemotherapy [17]. Second, ACH trials have suffered from a small number of enrolled subjects, difficult patient accrual, early termination of trials, and defects in the statistical methodology [25]. Most previous studies are underpowered to provide sufficient evidence to support the use of ACH in MIBC. Moreover, most RCTs have assessed the survival differences between ACH and observation (RC alone), so there have been few head-to-head trials comparing various ACH regimens. Thus, in terms of improving the survival outcomes in MIBC patients, a consensus on the optimal ACH regimen has yet to be reached.

In this meta-analysis of previous RCTs, we aimed to elucidate the efficacy of ACH and identify which ACH regimen was optimal in terms of survival benefit in MIBC patients who underwent RC. By direct pairwise meta-analysis, ACH correlated with a 36% improvement in PFS and a 21% improvement in OS compared with RC alone. These findings correspond well with the results of previous meta-analyses, which demonstrate that ACH can improve survival outcomes in MIBC [10, 16]. However, unlike previous meta-analyses, the main objective of our study was to determine the optimal ACH regimen for survival benefit by indirectly comparing various regimens [10, 16]. We used a network meta-analysis, which unlike a conventional pairwise meta-analysis, can provide an estimate of the relative efficacy between all interventions, even if some have never been compared head-to-head [26]. Based on the results of this network meta-analysis and rankograms of the various ACH regimens, the GCP combination was the most effective regimen for achieving significant improvements in PFS and OS. Notably, the GC and MAVC combinations, which are the most commonly used regimens in clinical practice, showed an inferior efficacy to RC alone in terms of survival benefit.

There are several strengths of our study. To the best of our knowledge, this is the first meta-analysis evaluating the optimal ACH regimen associated with survival benefit, which may help clinicians select an appropriate adjuvant chemotherapeutic regimen. Second, by only including prospectively designed RCTs in the meta-analysis, this study avoided the inherent bias due to heterogeneity. Third, the trials included in this study showed a relative consistency in the number of planned cycles (ranging from 3 to 4 cycles) and dose-specific ACH regimens.

Despite these strengths, the results drawn from this analysis should be cautiously interpreted due to several limitations. Because this meta-analysis pooled previously published trials, the association between ACH and survival outcomes could not be adjusted through multivariate analysis with other prognostic variables. Unknown or uncontrolled variables, such as inter-study variation in surgical technique (i.e., the quality of radical cystectomy and the extent of PLND), perioperative complications, chemotherapy-related toxicity, and patient dropout, may have affected the results of this study. Second, the current analysis incorporated studies performed over several decades (1990s, 2000s and 2010s), and the commonly used chemotherapeutic regimens changed over time; this might have influenced the baseline characteristics of MIBC patients, resulting in different survival outcomes. Furthermore, the definition of our primary endpoint, PFS, was inconsistent across trials. Some trials defined PFS as the time from RC, while others defined it as the time from randomization to the earliest occurrence of relapse or death from any cause. The time when patients were randomized also differed among studies, which may have led to minor differences in the PFS. Another limitation of this study was the difference in baseline disease severity. For example, a Spanish trial [21] enrolled pT3-4 or N+ patients only; the significant survival benefit of the GCP regimen may have been due to these patients having more advanced disease. Two recent RCTs [13, 14] did not show a survival benefit from the GC or MVAC regimen; in those studies, almost a third of patients (33%) or all patients had pT1-2 disease, respectively. Lastly, we only included studies that were published in English, which may have led to a language bias [27], although there was no evidence of publication bias in our analysis.

Although GC and MVAC combinations are both preferred ACH regimens in MIBC treatment, the GCP combination was the optimal regimen for survival benefit after RC based on the current network meta-analysis. However, additional well-designed, large-scale (enrollment of at least 1,000 subjects), prospective, randomized studies are required to verify the clinical efficacy of various ACH regimens, including the GCP combination, in MIBC.

## MATERIALS AND METHODS

The present analysis was conducted and reported based on the recommendations of the PRISMA guidelines [28].

### Search strategy

A literature search was conducted for all articles published in English until December 2016, using the Pubmed, Embase, and Cochrane Library databases. The following key words were used as search terms separately or in combination: (urothelial cancer OR urinary bladder OR bladder cancer OR bladder carcinoma) and (adjuvant chemotherapy OR post-operative chemotherapy) and (radical cystectomy). Conference abstracts were also included in this study if they met the eligibility criteria. Citation lists of all studies found were then used to identify other potentially relevant publications.

### Eligibility criteria

Based on the PRISMA guidelines, we used the Population, Intervention, Comparator, Outcome, and Study design system (PICOS) to define study eligibility [28]. The study population was defined as patients with MIBC, and the intervention was defined as various cisplatin-based ACH regimens. The comparator was RC alone, and the outcomes were PFS and OS. Only RCTs were included in this study.

Studies were included if they met the following inclusion criteria: (1) human research; (2) patients with MIBC who underwent RC; (3) cisplatin-based ACH; (4) reported outcome values (RFS and/or OS); (5) correlation between ACH and outcome values; (6) availability of Kaplan-Meier/uni- or multivariate Cox proportional hazard model results to estimate the HRs and their 95% CIs; and (7) RCTs. Exclusion criteria were: (1) letters, commentaries, case reports, reviews, and articles that did not provide raw data; (2) non-English articles; (3) studies using analyses other than survival analysis; and (4) carboplatin-based chemotherapy, or studies in which chemotherapy was delivered non-intravenously. If duplication of study populations or analyses of repeated data were identified, only the largest or most recent article was accepted. In studies that utilized both univariate and multivariate analyses to estimate clinical outcomes, the results of the multivariate analysis were used to estimate HRs and CIs. However, if inclusion of ACH in multivariate analysis was impossible due to negative results of the univariate analysis, the results of the univariate analysis were used. Two reviewers (HSK and CWJ) initially screened the relevant articles based on the titles and abstracts of all available literature. Next, full-text articles were independently examined by three reviewers (HSK, CK, and JHK) to determine whether they met the inclusion criteria. Any disagreements between reviewers were resolved by reaching a consensus with a fourth reviewer (HHK).

### Study quality assessments

Three reviewers (HSK, CWJ and JHK) independently estimated the methodological quality of each included study in accordance with the Reporting Recommendations for Tumor Marker Prognostic Studies (REMARK) guidelines [29]. Six items were assigned a score of 0 or 1, thus the final quality scale ranged from 0 (lowest) to 6 (highest). The three reviewers (HSK, CWJ and JHK) then compared the quality scores and reached a consensus value for each item.

### Data extraction

Two reviewers (HSK and JHK) independently extracted and crosschecked the required information from all eligible studies. Any conflicts in extracted data between the two authors were resolved by consensus. We did not contact authors of eligible studies for additional data. The required data were recorded according to the REMARK guidelines [29] as follows: (1) publication data: first author name, publication year, country, and period of recruitment; (2) characteristics of each study population: number of patients, median age, and gender distribution in the control and ACH groups; (3) tumor characteristics and pathologic tumor stage; and (4) treatment characteristics: regimen, dosage, planned cycles of ACH, and median follow-up period.

The primary endpoint of this study was PFS, and progression was defined as the subsequent occurrence of local recurrence, either at the operative site or in the regional lymph nodes, or distant metastasis after RC. The secondary endpoint was OS, defined as the interval between RC and death from MIBC or any other cause.

### Statistical analysis

A pairwise meta-analysis was conducted for direct comparison between control and ACH groups. Based on the methods depicted by DerSimonian and Laird for applying the inverse of variance as a weighting factor in random-effects models [30], the pooled HRs with 95% CIs were determined, indicating the impact of ACH regimens on each outcome (PFS and OS). The statistical evaluation for inter-study heterogeneity of the pooled HRs was performed using the Chi-squared-based Q and I-square tests [31]. Significant heterogeneity was defined by *p* < 0.1 for the *Q* test and > 50% for the I-square test among the selected studies. Sensitivity analysis was performed by evaluating the stability of the results after sequential omission of included studies.

Publication bias was assessed by graphical inspection of funnel plots, the Egger's test (linear regression analysis), and the Begg's test (rank correlation analysis) [32, 33]. Symmetrical inverted funnel plots indicated no significant publication bias; when bias is present, an inverted funnel plot should appear skewed and asymmetrical. Additionally, significant statistical publication bias was suspected when the *p*-values for the Begg's and Egger's tests were less than 0.05.

To indirectly compare the influence of each ACH regimen on the primary endpoint (PFS) and secondary endpoint (OS), we conducted a network meta-analysis using a Bayesian framework random effects model based on the Markov chain Monte Carlo algorithm known as Gibbs sampling, as implemented in WinBUGS 1.4 (MRC Biostatistics Unit, Cambridge, UK) [34]. The selection of a fixed or random effects model for reported outcomes was based on the deviance information criteria (DIC), which penalizes greater model complexity [35]. We modeled the binary outcomes for every ACH regimen group of every study and quantified the association between HRs with 95% CrIs among studies; CrIs can be regarded at similar to conventional CIs. Each analysis was based on noninformative priors for effect size and precision. We also examined inconsistency between direct and indirect estimates using a modified back-calculation approach [36]. The quality of the models was examined by inspecting convergence using Gelman-Rubin-Brooks plots, assessing autocorrelation between iterations of the Markov chain (MC), and determining whether the MC error was less than 5% of the posterior standard deviation.

The pairwise meta-analysis was performed using Review Manager v.5.1 (The Nordic Cochrane Center, The Cochrane Collaboration, Copenhagen, Denmark, 2008), and publication bias was estimated using R 2.13.0 (R development Core Team, Vienna, http://www.R-project.org). The network meta-analyses were conducted using R 3.2.2 (R development Core Team, Vienna, http://www.R-project.org) with the GeMTC package. All *p*-values were two-sided, and *p* < 0.05 was considered statistically significant except for the test of inconsistency, in which a one-sided *p* < 0.1 cutoff was adopted.

## SUPPLEMENTARY MATERIALS FIGURES AND TABLES



## Abbreviations

ACH: adjuvant chemotherapy
MIBC: muscle invasive bladder cancer
RC: radical cystectomy
PLND: pelvic lymph node dissection
RFS: recurrence free survival
RCT: randomized controlled trial
NACH: neoadjuvant chemotherapy
PRISMA: preferred reporting items for systematic reviews and meta-analyses
REMARK: reporting recommendations for tumor marker prognostic studies
C: cisplatin
CAP: cisplatin, doxorubicin and cyclophosphamide
CM: cisplatin and methotrexate
CMV: cisplatin, methotrexate and vinblastine
GC: gemcitabine and cisplatin
GCP: gemcitabine, cisplatin and paclitaxel
MVAC: methotrexate, vinblastine, doxorubicin and cisplatin
MVEC: methotrexate, vinblastine, epirubicin and cisplatin
PFS: progression-free survival
OS: overall survival
HR: hazard ratio
CI: confidence interval
CrI: credible interval.

## References

[1] Stein JP, Lieskovsky G, Cote R, Groshen S, Feng AC, Boyd S, Skinner E, Bochner B, Thangathurai D, Mikhail M (2001). Radical cystectomy in the treatment of invasive bladder cancer: long-term results in 1,054 patients. J Clin Oncol.

[2] Sternberg CN, Bellmunt J, Sonpavde G, Siefker-Radtke AO, Stadler WM, Bajorin DF, Dreicer R, George DJ, Milowsky MI, Theodorescu D, Vaughn DJ, Galsky MD, Soloway MS (2013). ICUD-EAU international consultation on bladder cancer 2012: chemotherapy for urothelial carcinoma-neoadjuvant and adjuvant settings. Eur Urol.

[3] Witjes JA, Comperat E, Cowan NC, De Santis M, Gakis G, Lebret T, Ribal MJ, Van der Heijden AG, Sherif A, European Association of U (2014). EAU guidelines on muscle-invasive and metastatic bladder cancer: summary of the 2013 guidelines. Eur Urol.

[4] Meeks JJ, Bellmunt J, Bochner BH, Clarke NW, Daneshmand S, Galsky MD, Hahn NM, Lerner SP, Mason M, Powles T, Sternberg CN, Sonpavde G (2012). A systematic review of neoadjuvant and adjuvant chemotherapy for muscle-invasive bladder cancer. Eur Urol.

[5] Grossman HB, Natale RB, Tangen CM, Speights VO, Vogelzang NJ, Trump DL, deVere White RW, Sarosdy MF, Wood DP, Raghavan D, Crawford ED (2003). Neoadjuvant chemotherapy plus cystectomy compared with cystectomy alone for locally advanced bladder cancer. N Eng J Med.

[6] Kitamura H, Tsukamoto T, Shibata T, Masumori N, Fujimoto H, Hirao Y, Fujimoto K, Kitamura Y, Tomita Y, Tobisu K (2014). Randomised phase III study of neoadjuvant chemotherapy with methotrexate, doxorubicin, vinblastine and cisplatin followed by radical cystectomy compared with radical cystectomy alone for muscle-invasive bladder cancer: Japan Clinical Oncology Group Study JCOG0209. Ann Oncol.

[7] Winquist E, Kirchner TS, Segal R, Chin J, Lukka H (2004). Neoadjuvant chemotherapy for transitional cell carcinoma of the bladder: a systematic review and meta-analysis. J Urol.

[8] Collaboration ABCAM-a. (2005). Neoadjuvant chemotherapy in invasive bladder cancer: update of a systematic review and meta-analysis of individual patient data advanced bladder cancer (ABC) meta-analysis collaboration. Eur Urol.

[9] Freiha F, Reese J, Torti FM (1996). A randomized trial of radical cystectomy versus radical cystectomy plus cisplatin, vinblastine and methotrexate chemotherapy for muscle invasive bladder cancer. J Urol.

[10] Vale CL (2005). Adjuvant chemotherapy in invasive bladder cancer: a systematic review and meta-analysis of individual patient data: Advanced Bladder Cancer (ABC) meta-analysis collaboration. Eur Urol.

[11] Lehmann J, Retz M, Wiemers C, Beck J, Thuroff J, Weining C, Albers P, Frohneberg D, Becker T, Funke PJ, Walz P, Langbein S, Reiher F (2005). Adjuvant cisplatin plus methotrexate versus methotrexate, vinblastine, epirubicin, and cisplatin in locally advanced bladder cancer: results of a randomized, multicenter, phase III trial (AUO-AB 05/95). J Clin Oncol.

[12] Lehmann J, Franzaring L, Thuroff J, Wellek S, Stockle M (2006). Complete long-term survival data from a trial of adjuvant chemotherapy vs control after radical cystectomy for locally advanced bladder cancer. BJU Int.

[13] Stadler WM, Lerner SP, Groshen S, Stein JP, Shi SR, Raghavan D, Esrig D, Steinberg G, Wood D, Klotz L, Hall C, Skinner DG, Cote RJ (2011). Phase III study of molecularly targeted adjuvant therapy in locally advanced urothelial cancer of the bladder based on p53 status. J Clin Oncol.

[14] Cognetti F, Ruggeri EM, Felici A, Gallucci M, Muto G, Pollera CF, Massidda B, Rubagotti A, Giannarelli D, Boccardo F (2012). Adjuvant chemotherapy with cisplatin and gemcitabine versus chemotherapy at relapse in patients with muscle-invasive bladder cancer submitted to radical cystectomy: an Italian, multicenter, randomized phase III trial. Ann Oncol.

[15] Sternberg CN, Skoneczna I, Kerst JM, Albers P, Fossa SD, Agerbaek M, Dumez H, de Santis M, Theodore C, Leahy MG, Chester JD, Verbaeys A, Daugaard G (2015). Immediate versus deferred chemotherapy after radical cystectomy in patients with pT3-pT4 or N+ M0 urothelial carcinoma of the bladder (EORTC 30994): an intergroup, open-label, randomised phase 3 trial. Lancet Oncol.

[16] Leow JJ, Martin-Doyle W, Rajagopal PS, Patel CG, Anderson EM, Rothman AT, Cote RJ, Urun Y, Chang SL, Choueiri TK, Bellmunt J (2014). Adjuvant chemotherapy for invasive bladder cancer: a 2013 updated systematic review and meta-analysis of randomized trials. Eur Urol.

[17] Skinner DG, Daniels JR, Russell CA, Lieskovsky G, Boyd SD, Nichols P, Kern W, Sakamoto J, Krailo M, Groshen S (1991). The role of adjuvant chemotherapy following cystectomy for invasive bladder cancer: a prospective comparative trial. J Urol.

[18] Studer UE, Bacchi M, Biedermann C, Jaeger P, Kraft R, Mazzucchelli L, Markwalder R, Senn E, Sonntag RW (1994). Adjuvant cisplatin chemotherapy following cystectomy for bladder cancer: results of a prospective randomized trial. J Urol.

[19] Bono A, Benvenuti C, Gibba A, Guazzeri S, Cosciani-Cunico S, Anselmo G, Martini E, Parma G, Ferrari P, Viggiano G (1997). Adjuvant chemotherapy in locally advanced bladder cancer. Final analysis of a controlled multicentre study. Acta Urologica Italica.

[20] Otto T, Börgemann C, Krege S (2001). Adjuvant chemotherapy in locally advanced bladder cancer (PT3/PN1-2, M0)—a phase III study [abstract]. Eur Urol.

[21] Paz-Ares L, Solsona E, Esteban E, Saez A, Gonzalez-Larriba J, Anton A, Hevia M, de la Rosa F, Guillem V, Bellmunt J (2010). Randomized phase III trial comparing adjuvant paclitaxel/gemcitabine/cisplatin (PGC) to observation in patients with resected invasive bladder cancer: results of the Spanish Oncology Genitourinary Group (SOGUG) 99/01study [abstract LBA4518]. J Clin Oncol.

[22] Park J, Park S, Song C, Doo C, Cho YM, Ahn H, Kim CS (2007). Effectiveness of adjuvant chemotherapy in transitional cell carcinoma of the urinary bladder with lymph node involvement and/or lymphovascular Invasion treated by radical cystectomy. Urology.

[23] Yelfimov DA, Frank I, Boorjian SA, Thapa P, Cheville JC, Tollefson MK (2014). Adjuvant chemotherapy is associated with decreased mortality after radical cystectomy for locally advanced bladder cancer. World J Urol.

[24] Kim HS, Piao S, Moon KC, Jeong CW, Kwak C, Kim HH, Ku JH (2015). Adjuvant chemotherapy correlates with improved survival after radical cystectomy in patients with pT3b (macroscopic perivesical tissue invasion) bladder cancer. J Cancer.

[25] Suttmann H, Kamradt J, Lehmann J, Stockle M (2007). Improving the prognosis of patients after radical cystectomy. Part II: the role of perioperative chemotherapy. BJU Int.

[26] Mills EJ, Thorlund K, Ioannidis JP (2013). Demystifying trial networks and network meta-analysis. BMJ.

[27] Egger M, Zellweger-Zähner T, Schneider M, Junker C, Lengeler C, Antes G (1997). Language bias in randomised controlled trials published in English and German. Lancet.

[28] Moher D, Liberati A, Tetzlaff J, Altman DG (2009). Preferred reporting items for systematic reviews and meta-analyses: the PRISMA statement. Ann Intern Med.

[29] Altman DG, McShane LM, Sauerbrei W, Taube SE (2012). Reporting recommendations for tumor marker prognostic studies (REMARK): explanation and elaboration. BMC Med.

[30] DerSimonian R, Laird N (1986). Meta-analysis in clinical trials. Control Clin Trials.

[31] Higgins JP, Thompson SG, Deeks JJ, Altman DG (2003). Measuring inconsistency in meta-analyses. BMJ.

[32] Begg CB, Mazumdar M (1994). Operating characteristics of a rank correlation test for publication bias. Biometrics.

[33] Egger M, Smith GD, Schneider M, Minder C (1997). Bias in meta-analysis detected by a simple, graphical test. BMJ.

[34] Lu G, Ades AE (2004). Combination of direct and indirect evidence in mixed treatment comparisons. Stat Med.

[35] Caldwell DM, Ades AE, Higgins JP (2005). Simultaneous comparison of multiple treatments: combining direct and indirect evidence. BMJ.

[36] Dias S, Welton NJ, Caldwell DM, Ades AE (2010). Checking consistency in mixed treatment comparison meta-analysis. Stat Med.

