# Supplementary material for: Adjuvant chemotherapy for muscle-invasive bladder cancer: a systematic review and network meta-analysis of randomized clinical trials
Source: Oncotarget. 2017 Sep 18;8(46):81204–14. doi: 10.18632/oncotarget.20979 (PMC5655275; doi:10.18632/oncotarget.20979)
Supplement: Supplementary file 1 [file oncotarget-08-81204-s001.pdf]

Adjuvant chemotherapy for muscle-invasive bladder cancer: a systematic review and network meta-analysis of randomized clinical trials

SUPPLEMENTARY MATERIALS

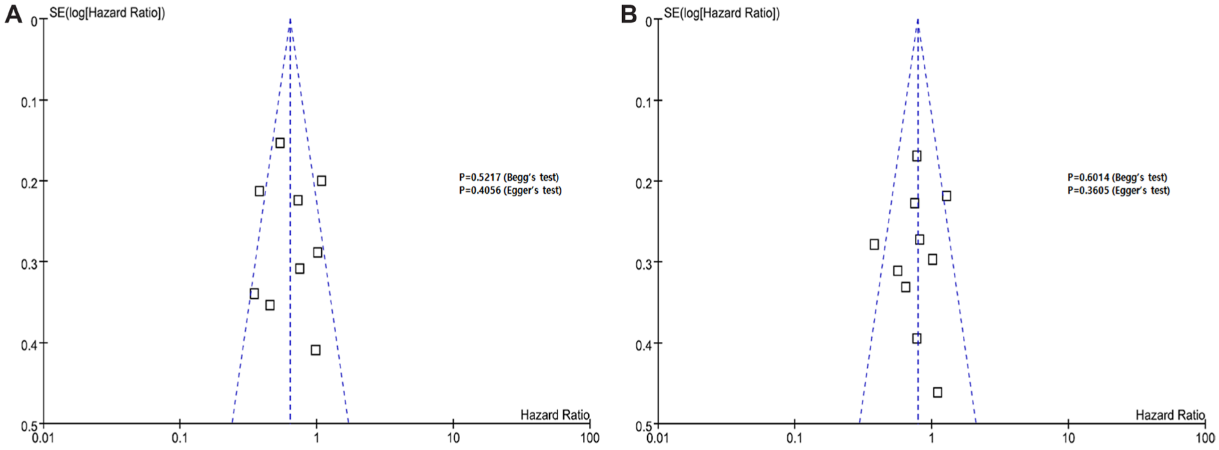

Supplementary Figure 1: Funnel plots for publication bias test of prognosis. Each point represents a separate study for the indicated association. Vertical line represents the mean effect size. (**A**) progression-free survival; (**B**) overall survival.

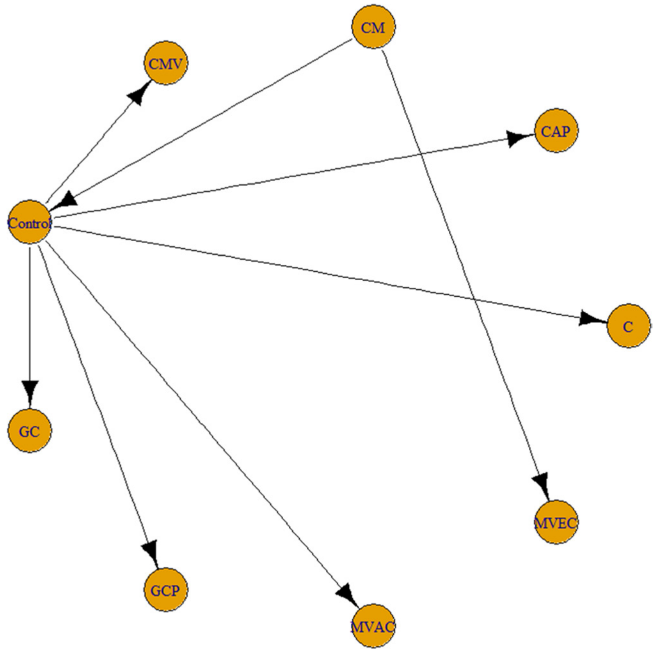

Supplementary Figure 2: Network geometry of clinical trials of adjuvant chemotherapy for progression-free survival in muscle-invasive bladder cancer. Lines represent direct comparison trials. C: cisplatin; CAP: cisplatin, doxorubicin, and cyclophosphamide; CM: cisplatin and methotrexate; CMV: cisplatin, methotrexate, and vinblastine; GC: gemcitabine and cisplatin; GCP: gemcitabine, cisplatin, and paclitaxel; MVAC: methotrexate, vinblastine, doxorubicin, and cisplatin; MVEC: methotrexate, vinblastine, epirubicin, and cisplatin.

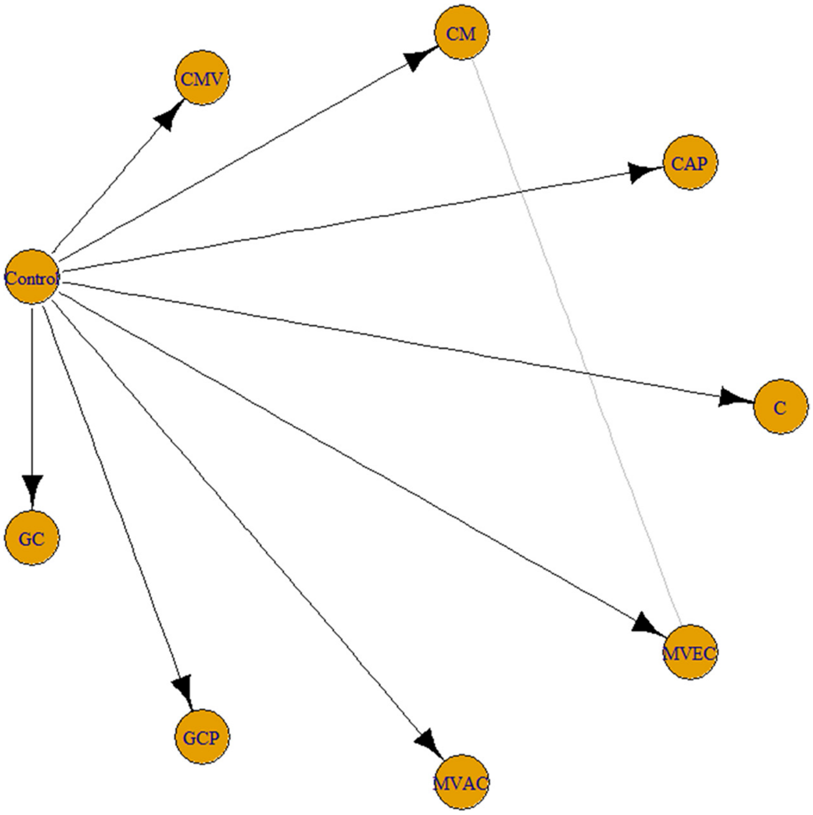

Supplementary Figure 3: Network geometry of clinical trials of adjuvant chemotherapy for overall survival in muscle-invasive bladder cancer. Lines represent direct comparison trials. C: cisplatin; CAP: cisplatin, doxorubicin, and cyclophosphamide; CM: cisplatin and methotrexate; CMV: cisplatin, methotrexate, and vinblastine; GC: gemcitabine and cisplatin; GCP: gemcitabine, cisplatin, and paclitaxel; MVAC: methotrexate, vinblastine, doxorubicin, and cisplatin; MVEC: methotrexate, vinblastine, epirubicin, and cisplatin.

Supplementary Table 1: Ranking of progression-free survival (% of 5,000 iterations)

Rank	GC	Control	C	MVAC	CM	CAP	MVEC	CMV	GCP
1	0.0	0.0	0.1	1.1	0.4	0.7	0.5	30.5	62.1
2	0.2	0.0	1.0	3.5	3.7	7.2	15.7	38.8	29.9
3	1.4	0.0	0.5	9.7	14.0	23.5	28.5	12.3	5.6
4	3.6	1.8	7.5	8.4	26.7	18.9	21.5	9.6	2.0
5	6.8	7.3	10.8	10.6	22.4	24.6	12.2	5.0	0.4
6	13.2	22.4	15.0	12.8	13.4	14.5	6.7	2.1	0.0
7	19.3	35.9	14.5	10.5	8.3	5.8	4.9	0.8	0.0
8	26.3	26.2	19.8	13.6	6.5	3.1	3.8	0.6	0.0
9	29.3	6.3	26.3	29.9	4.7	1.5	1.7	0.4	0.0

Lower rank indicates greater survival benefit.

Supplementary Table 2: Ranking of overall survival (% of 5,000 iterations)

Rank	GC	MVAC	Control	C	CMV	CM	CAP	MVEC	GCP
1	0.0	1.7	0.0	0.4	6.0	0.6	0.2	0.2	87.6
2	0.3	8.5	0.0	4.9	23.8	8.5	22.0	23.4	8.7
3	0.7	6.0	0.6	6.8	12.0	22.7	19.4	29.8	2.2
4	1.3	6.7	3.7	8.2	11.6	25.1	19.4	22.9	1.1
5	3.2	8.0	14.0	11.7	11.5	21.5	17.3	12.6	0.4
6	5.6	8.9	32.7	14.6	10.1	11.3	11.0	5.7	0.1
7	13.4	10.7	34.0	18.2	9.3	6.3	5.6	2.6	0.0
8	33.6	16.9	13.5	19.9	9.0	3.2	3.1	0.9	0.0
9	42.0	32.7	1.5	15.4	6.6	0.9	0.7	0.2	0.0

Lower rank indicates greater survival benefit.
